# Gadolinium-Based Paramagnetic Relaxation Enhancement Agent Enhances Sensitivity for NUS Multidimensional NMR-Based Metabolomics

**DOI:** 10.3390/molecules26175115

**Published:** 2021-08-24

**Authors:** Chandrashekhar Honrao, Nathalie Teissier, Bo Zhang, Robert Powers, Elizabeth M. O’Day

**Affiliations:** 1 Olaris, Inc., Waltham, MA 02451, USA; chonrao@olarisbor.com (C.H.); nteissier@olarisbor.com (N.T.); bozhangchem@gmail.com (B.Z.); 2 Department of Chemistry, University of Nebraska-Lincoln, Lincoln, NE 68588, USA; 3 Nebraska Center for Integrated Biomolecular Communication, University of Nebraska-Lincoln, Lincoln, NE 68588, USA

**Keywords:** NMR, metabolomics, paramagnetic, relaxation, gadolinium

## Abstract

Gadolinium is a paramagnetic relaxation enhancement (PRE) agent that accelerates the relaxation of metabolite nuclei. In this study, we noted the ability of gadolinium to improve the sensitivity of two-dimensional, non-uniform sampled NMR spectral data collected from metabolomics samples. In time-equivalent experiments, the addition of gadolinium increased the mean signal intensity measurement and the signal-to-noise ratio for metabolite resonances in both standard and plasma samples. Gadolinium led to highly linear intensity measurements that correlated with metabolite concentrations. In the presence of gadolinium, we were able to detect a broad array of metabolites with a lower limit of detection and quantification in the low micromolar range. We also observed an increase in the repeatability of intensity measurements upon the addition of gadolinium. The results of this study suggest that the addition of a gadolinium-based PRE agent to metabolite samples can improve NMR-based metabolomics.

## 1. Introduction

Metabolomics is a rapidly expanding field that relies on the detection and quantification of small molecular-weight (MW < 1500 Daltons) compounds present in a biological sample. Metabolite levels are often correlated with different disease states or phenotypic outcomes, which can lead to the development of highly valuable biomarkers and provide novel insights into human health and disease [1,2,3,4,5,6,7]. Nuclear magnetic resonance (NMR) spectroscopy has proven to be a powerful tool for metabolomics that meets the analytical requirements needed to achieve a robust and accurate characterization of the metabolome [8,9,10,11]. Conventional NMR-based approaches rely on one-dimensional (1D) ^1^H NMR experiments, which can facilitate the absolute quantification of metabolites. However, chemical shift overlap may limit the number of metabolites that can be accurately measured, which often relies on the application of peak-fitting algorithms. The size and completeness of the reference database used by these peak fitting algorithms will also limit the number of metabolites that can be quantified. Multi-dimensional techniques such as two-dimensional (2D) ^1^H-^13^C Heteronuclear Single Quantum Correlation (HSQC) spectroscopy can increase resolution by dispersing the chemical shifts along the carbon dimension, but necessitates long acquisition times due to the low natural abundance of ^13^C (1.1%), thus limiting the real-world practicality of this approach [12].

Expanding upon the work of Rai [13] and Von Schlippenbach [14], we recently demonstrated that non-uniform sampling (NUS) can be used to reduce the acquisition time of a 2D ^1^H-^13^C HSQC experiment to empower semi-quantitative metabolomics [15]. Indeed, a one-hour experiment using a 25% NUS ^1^H-^13^C HSQC led to 4-fold improvement in sensitivity, which also yielded highly linear and repeatable data. Further, we established guidelines based on a signal-to-noise ratio (S/N) to enable the reliable detection of a broad range of metabolites in the low micromolar range with a coefficient of variation (CV) of less than 20%. Using these results as our baseline, we sought herein to systematically evaluate the effects of relaxation delays in combination with paramagnetic relaxation enhancement (PRE) agents to further improve the sensitivity of 2D NMR experiments for metabolomics. First, we improved the mean signal intensity and S/N of a 25% NUS ^1^H-^13^C HSQC experiment by optimizing the relaxation delay and the number of scans. Then, we observed that the addition of a gadolinium-based PRE agent further improved the S/N of the 25% NUS ^1^H-^13^C HSQC spectra for both a model mixture and plasma samples. A lower limit of detection and quantification was achieved for most metabolites, but the most dramatic improvement in signal intensity was seen for the weakest peaks. We also observed that the addition of the PRE agent maintained linearity for all metabolites over a concentration range from 50 μM to 2 mM. These intensity measurements were highly repeatable, leading to smaller CVs. Overall, our results demonstrate that PRE agents can improve the sensitivity of 2D NUS NMR spectra routinely used in metabolomic studies.

## 2. Results

### 2.1. Optimizing the Relaxation Delay for Semi-Quantitative Metabolomics

A fundamental principle of NMR spectroscopy is that increasing the number of experimental scans (N) increases the S/N ratio by a factor of √N [16]. For pulsed NMR experiments, the relaxation delay, commonly known as d1, is the time required between scans to allow spins to return to equilibrium. The optimal d1 time depends on the longitudinal relaxation (T_1_) rate—the time required for full restoration of the nuclear spin to equilibrium along the direction of the polarizing magnetic field [16]. Each nuclei in a molecule has a different T_1_ value, and for small molecules like metabolites T_1_ values can be several seconds long. For example, formate has a T_1_ > 9 s at 600 MHz [17]. For quantitative NMR, it is advised to set d1 to 5 × T_1_ of the slowest relaxing nuclei in a sample [17]. This would require a d1 of upwards of a minute in length, leading to impractically long acquisition times that are not feasible for high-throughput NMR metabolomics. In practice, d1 is commonly set to a pre-determined value that allows for a relative quantitative comparison between spectra collected under identical conditions. It is important to note that only a comparison between the same metabolite can be made in this manner across the spectral dataset. A comparison between two or more different metabolites would be meaningless because of the d1-dependent variation in peak integrals that distorts the relationship between peak integral and metabolite concentration.

**Figure 1:**
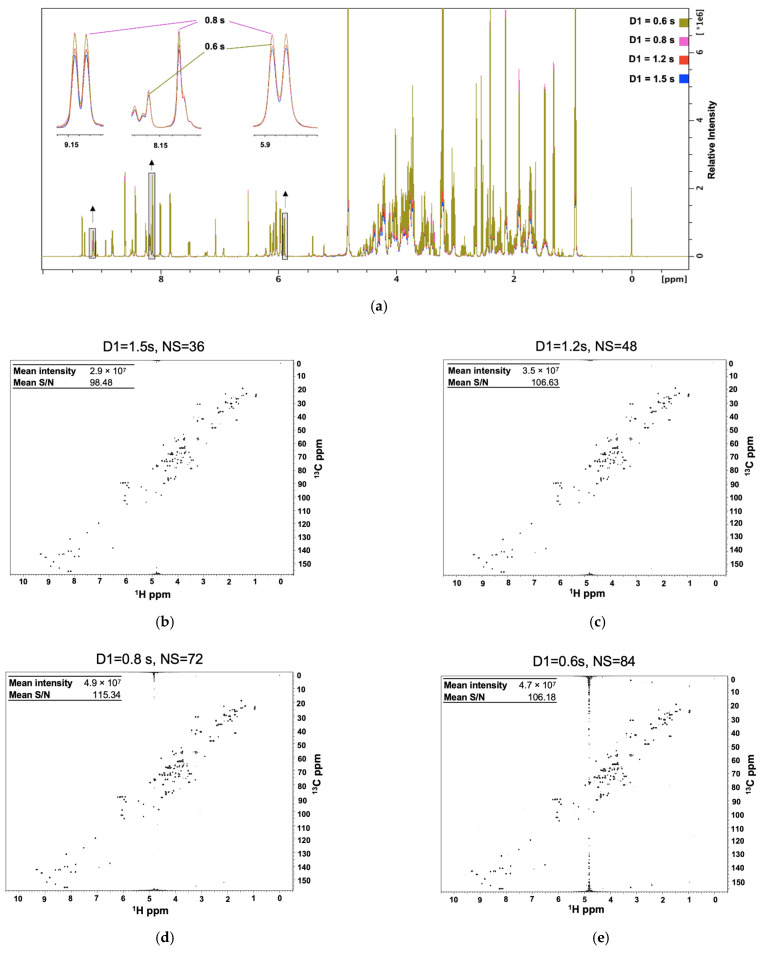
An optimized combination of relaxation delay (d1) and number of scans (N) improved the S/N for (**a**) 1D ^1^H and (**b**–**e**) 2D ^1^H-^13^C HSQC spectra of a model mixture of metabolites in time equivalent experiments. Four 1D ^1^H NMR spectra are overlaid and color-coded according to the d1 value: 1.5 s (blue), 1.2 s (red), 0.8 s (green), 0.6 s (purple). The boxed regions in the 1D ^1^H NMR spectra are shown as expanded inserts above each corresponding arrow.

A model mixture (“Reference 1”) was composed of 29 commonly observed human metabolites, which included amino acids, organic acids, biogenic amines, sugars, etc., from the literature [18,19,20,21] as well as metabolites commonly observed in our own clinical studies. To find the optimal d1 for a model mixture of 29 metabolites (Reference 1), we recorded time equivalent experiments (4 min ± 8 s) with varying d1 values of 1.5 s, 1.2 s, 0.8 s and 0.6 s and observed the changes in both the 1D ^1^H NMR spectra and 2D 25% NUS ^1^H-^13^C HSQC spectra (Figure 1). At first, as the d1 decreased, the signal intensity for the majority of the metabolites increased, which is expected due to the increased number of scans (N = 64 to 92 for the 1D- and N = 36 to 84 for the 2D-experiments). For the 25% NUS ^1^H-^13^C HSQC spectra, we observed a steady increase in the mean intensity of metabolites from 2.9 × 10^7^ to 4.9 × 10^7^ as d1 decreased from 1.5 s to 0.8 s. Similarly, the mean S/N increased from 98.48 to 115.34. However, the mean S/N and intensity reached a maximum at a d1 of 0.8 s. As evident by the expanded regions of the 1D ^1^H NMR spectra (Figure 1a), peak intensities began to decrease at a d1 of 0.6 s despite the larger number of scans. This is consistent with the 25% NUS ^1^H-^13^C HSQC spectra at a d1 of 0.6 s, where the mean S/N and intensity decreased to 106.18 and 4.7 × 10^7^, respectively. Furthermore, significant solvent artifacts were observed in the HSQC spectra relative to longer d1 values. Presumably, at a d1 of 0.6 s, factors related to T_1_ dominate spectral sensitivity, which could not be negated by the allowed increase in the number of scans. This led us to select 0.8 s as the optimal d1 value for improved S/N.

### 2.2. Gadolinium Provides Enhanced Sensitivity

The addition of PRE agents has been previously used to accelerate NMR data acquisition [13,17,22,23,24,25]. PRE agents contain unpaired electrons and decrease T_1_ relaxation times for all nuclei in a sample due to dipolar interactions between nuclear and electron spin states. The PRE effect is very large, owing to the large magnetic moment of an unpaired electron, and can be tunable by adjusting the concentration of the PRE agent [17]. By combining NUS with the relaxation enhancing agent, Cu(EDTA), Rai and colleagues demonstrated a 22-fold reduction in the 2D ^1^H-^13^C HSQC data collection time to quantify a handful of urine metabolites [13]. Gadolinium-based contrast agents have been widely used in MRI diagnostic imaging, for studying soluble proteins, for characterizing protein-protein, protein-oligosaccharides, and protein-nucleic acid complexes, and for investigating membrane proteins using NMR spectroscopy [26,27]. Sakol et al. have also shown the utility of the Gd-based contrast agent, Gd-DOTA, for cellular localization studies using NMR spectroscopy [28]. Similarly, Mulder and colleagues utilized gadolinium-based PRE agents and achieved a 3- to 4-fold improvement in acquisition time for quantifying several plasma metabolites [17]. We sought to expand upon these findings by focusing on parameters to increase spectral sensitivity for a fixed-time experiment (1 h ± 4 min) instead of accelerating acquisition times.

**Figure 2:**
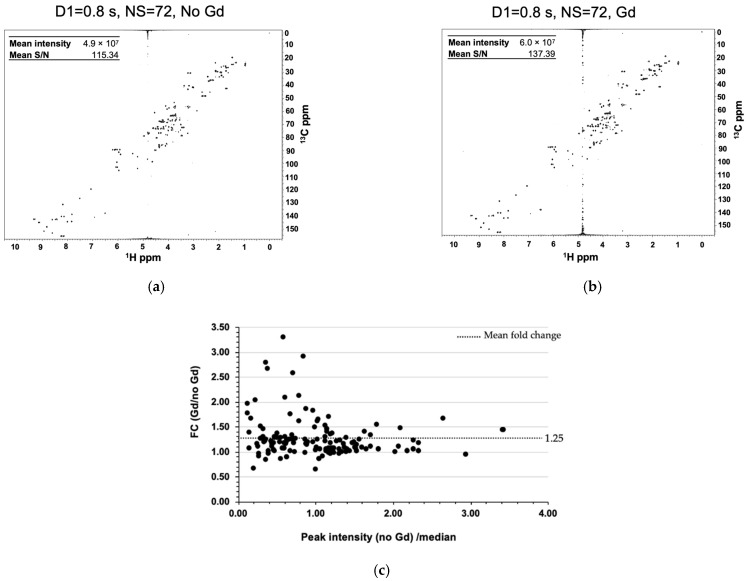
Gadolinium improves S/N and mean intensity of metabolite resonances. 2D ^1^H-^13^C HSQC spectra (**a**) without and (**b**) with the addition of Gd. (**c**) Fold change of the median normalized peak intensity in the presence of Gd.

We first assessed the 1D ^1^H spectral changes for Reference 1 (Supplementary Material Table S1) with a d1 of 0.8 s with increasing concentrations (0.25 mM to 1 mM) of Cu (EDTA) and Gadobutrol (Gd) (Figure S1), a gadolinium-containing macrocyclic that has previously been shown to enhance the relaxation rates of urine metabolites [17,25] (Figure S2). In general, contrast agents containing Gd shorten T_1_ and T_2_ relaxation rates through a dipole–dipole interaction between the unpaired electron of Gd and nuclei in the compound. The decrease in T_1_ and T_2_ rates depends on the contrast agent used and its concentration, the charge state of the compound, the viscosity of the solution, and the protein affinity of either the compound or contrast agent, among other issues. The typical range of T_1_ values for nuclei of common metabolites such as glucose, lactate, citrate, acetate, glutamine, and alanine are between 0.9 and 4 s. Similarly, T_2_ values range from 100 to 600 ms [17,28,29]. In the presence of Gd, T_1_ values can decrease from 2- to 10-fold depending on the concentration of Gd. A similar reduction is observed for T_2_, but is more pronounced at higher Gd concentrations. Accordingly, NMR resonances will significantly broaden into the baseline with the increase in Gd concentration [28]. Experimentally, we observed that a concentration of Gd at 0.25 mM allowed us to decrease our recycle delay to 0.8 s and achieve an overall increase in sensitivity while avoiding substantial line broadening. As the concentration of the Gd agent increased, the decrease in T_2_ and the associated peak broadening eventually eclipsed the reduction in T_1_ and negated any intensity gains from a larger number of scans [17,28,30,31]. In agreement with these observations, at 0.25 mM Gd, we noted an increase in intensities for the majority of metabolite resonances. As the concentration of Gd increased to 0.5 mM, a handful of metabolite resonances continued to show an increase in intensity, while others began to broaden. At 1 mM Gd, the majority of resonances were diminished compared to the control that lacked Gd. Interestingly, our results are in line with the theoretical optimal recycle delay predictions of Rovnyak et al. [32]. To perform the comparison, we identified NMR relaxation times reported in the literature for metabolites included in our study. For example, the work by Mulder et al. [17] demonstrated that the addition of Gd at a concentration of 0.5 mM to a mixture of small molecules (glucose, creatinine, citrate, glutamine, acetate, alanine, etc.) greatly reduced the T_1_ relaxation times by 2- to 10-fold, resulting in an average T_1_ relaxation time of ~0.6 s. Using the equation derived from Rovnyak et al., in the presence of Gd the theoretical optimal recycle delay would be ~0.8 s (1.26 × 0.6 s), which is in perfect agreement with our experimental findings of an optimal d1 of 0.8 s. In the presence of Cu (EDTA), we observed a decrease in NMR resonance intensities and significant line broadening at all concentrations tested. These results suggest that the addition of 0.25 mM of Gd may offer an optimal improvement in S/N. Indeed, when we recorded a 25% NUS ^1^H-^13^C HSQC spectrum with a d1 of 0.8 s in the presence of 0.25 mM Gd, we observed an increase in both the mean intensity and mean S/N for Reference 1 (Figure 2). While the average fold-change increase in peak intensity due to the addition of Gd was modest (1.25-fold), we observed large fold-change increases (>2-fold) for the lowest intensity resonances (Figure 2c). Thus, the addition of Gd could improve the ability to detect low abundant metabolites. Of note, significant differences were observed in the intensity for individual metabolites, suggesting that Gd affects each metabolite to a different extent. Previous studies have suggested that a charge distribution, especially anionic metabolites, may be more affected by Gd [22,24]. We also verified that, for 0.25 mM Gd, the optimal d1 remained at 0.8 s as measured by both an increase in mean peak intensity and mean S/N (Figure S3). Taken together, our results suggest that the addition of Gd can improve both S/N and peak intensities, which will result in an overall sensitivity improvement, leading to a higher accuracy and precision in the measurement of metabolite concentrations.

### 2.3. Gadolinium Maintains Linearity

**Figure 3:**
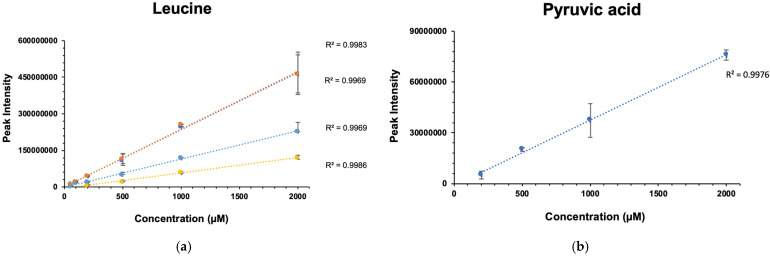
Linear regression analysis of NMR resonance intensities for (**a**) leucine and (**b**) pyruvic acid peaks.

Metabolomics requires quantification across a broad range of concentrations and the ability to accurately detect changes in metabolite levels [33,34]. We previously demonstrated that NUS ^1^H-^13^C HSQC metabolite profiling is highly linear in the 0.05 μM to 2 mM range [15]. Rai and colleagues also observed that the addition of a PRE agent, Cu(EDTA), maintained linearity for an NUS ^1^H-^13^C HSQC experiment that measured four amino acids (glycine, alanine, valine and methionine) over a concentration range of 24 to 78 mM [13]. We first sought to confirm that the addition of Gd maintained linearity over a broad concentration range. A series of six NUS ^1^H-^13^C HSQC spectra were recorded for a mixture containing 29 metabolites (Reference 2) with concentrations ranging from 50 μM to 2 mM (Table S2). For each NMR resonance, the peak intensity was plotted as a function of concentration and the data were fit to a linear regression model (Figure S4). Example plots of the four NMR resonance peaks for leucine and the single resonance peak for pyruvic acid are shown in Figure 3. More than 98% of the metabolite resonances displayed a correlation coefficient of R^2^ > 0.9, indicating excellent linearity (Table 1). Interestingly, glucose resonances, which can be affected by isomers and conformational changes, had an R^2^ > 0.99 that was an improvement from our previous findings without Gd, where we observed an R^2^ of ~0.8 [15]. Overall, this analysis demonstrated that peak intensities are highly linear as a function of metabolite concentration for NUS ^1^H-^13^C HSQC spectra in the presence of Gd.

### 2.4. Gadolinium Improves the Lower Limit of Detection and Quantification

We next sought to determine the lower limit of detection (LOD) and lower limit of quantification (LOQ) for our NUS measurements in the presence of Gd. LOD and LOQ are defined as follows:

LOD = 3 × σ(1)

LOQ = 10 × σ(2)

where the variance of the noise (σ) was estimated by the median absolute deviation (*MAD*). *MAD* was calculated from the COLMAR database [35], where the positive values for all non-peak data (*X_i_*) were used in the following equations:

*MAD* = *median_i_*(|*X_i_* − *median_i_*(*X_j_*)|) (3)

σ = 1.4826 × MAD(4)

Table 2 and Table 3 list the LOD and LOQ for each of the resonances detected in Reference 2. Metabolites with multiple resonances have an LOD/LOQ for each observed peak, and thus metabolites with multiple peaks will have a range of LOD/LOQ values. The average LOD and LOQ in the presence of Gd was 7.8 ± 0.3 μM and 26 ± 1 μM, respectively. This is a dramatic improvement over our previous findings that yielded an average LOD and LOQ of 19.1 μM and 65.6 μM, respectively [15]. These prior NMR experiments lacked the addition of Gd and used a longer d1 of 1.5 s. Thus, it is possible to detect lower abundant metabolites by adding Gd and decreasing d1. We also compared the effects of different NMR probes on LOD/LOQ. For the same d1 of 1.5 s, a TCI helium-cooled probe had a lower LOD/LOQ compared to a TXI nitrogen-cooled probe (Table S3).

### 2.5. Gadolinium Maintains Reproducibility

**Figure 4:**
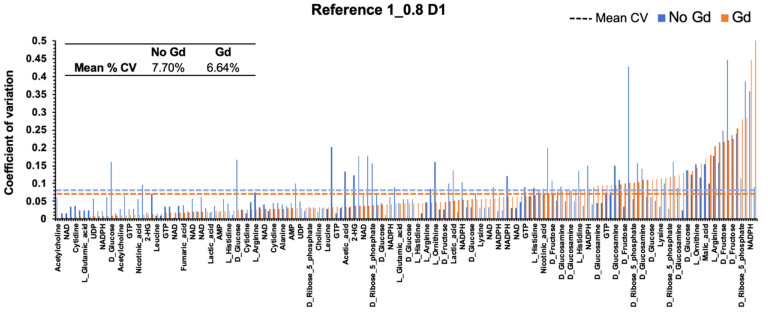
Gadolinium maintains the reproducibility of NMR intensity measurements. The percent coefficient of variation (%CV) in the peak intensities measured from NUS ^1^H-^13^C HSQC spectra. The %CV decreased for metabolite resonances in the presence of Gd (orange) compared to no Gd (blue).

Highly reproducible measurements are required to detect changes in the large number of samples associated with metabolomics studies. We previously demonstrated that intensity measurements from NUS ^1^H-^13^C HSQC experiments with a d1 of 1.5 s were highly reproducible as evident by a percent coefficient of variation (%CV) of 14 ± 9% for a model mixture containing 15 metabolites at a concentration of 500 μM [15]. By decreasing the d1 to 0.8 s, we observed a decrease in the %CV to 8 ± 8% (Figure 4) for three replicates of Reference 1. This was expected, given that the increased number of scans would lead to an increase in peak intensities. We only observed a modest decrease in %CV to 7 ± 7% (Figure 4) by adding Gd to the samples while maintaining a d1 of 0.8 s. This suggests that the addition of Gd does not negatively impact the reproducibility of NUS ^1^H-^13^C HSQC experiments and may increase the reliability of these measurements.

### 2.6. Gadolinium Effect on Plasma Metabolites

**Figure 5:**
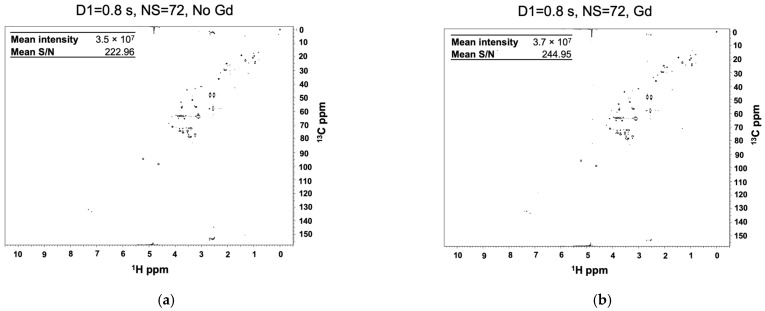

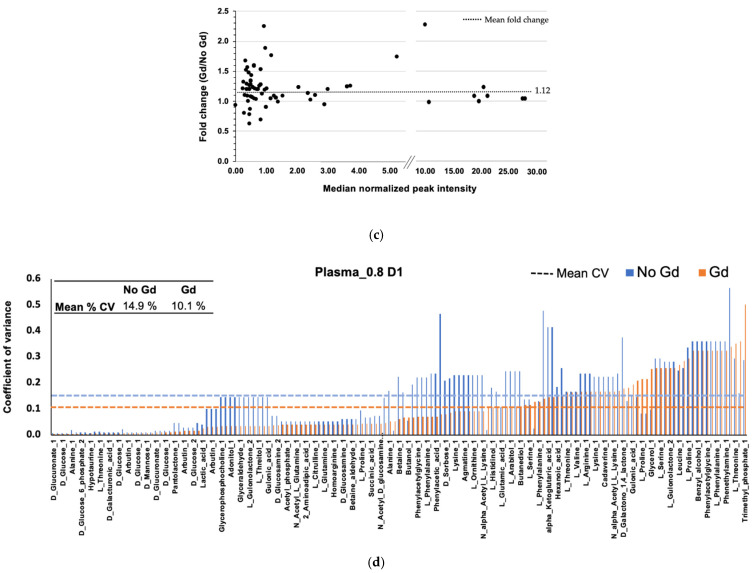
Gadolinium improves S/N and the mean intensity of plasma metabolite resonances. ^1^H-^13^C HSQC spectra (**a**) without and (**b**) with the addition of gadolinium. (**c**) Fold change of median normalized intensity in the presence of gadolinium. (**d**) Percent coefficient of variation (% CV) measured intensity decreases for metabolite resonances in the presence of Gd (orange) compared to no Gd (blue).

We next assessed the effects of Gd on our ability to detect and quantify metabolites using a commercially available standard pooled human plasma sample. We recorded a 25% NUS ^1^H-^13^C HSQC with or without the addition of Gd, and with a relaxation delay of 0.8 s, a constant scan number of 72, and an acquisition time of ~1 h. The addition of Gd led to a 1.12-fold increase in overall mean peak intensities. This increase was slightly less pronounced than the fold change of 1.25 observed with the model mixture and could be due to the presence of additional anions and salts, which are known to influence the impact of PRE agents [22,24]. Nonetheless, as noted for the model mixtures, we observed that the largest increase in fold change was associated with low-intensity resonances. These results further suggest that the addition of Gd could improve our ability to detect low abundant metabolites (Figure 5c). Furthermore, the % CV was lowered from 15% to 10% for the pooled human plasma sample in the presence of Gd (Figure 5d). Collectively, these results suggest that the addition of Gd to plasma samples increases the S/N for metabolite NMR resonances, especially for low abundant metabolites, and increases the reproducibility of intensity measurements. Overall, the addition of Gd to a metabolomics sample could facilitate an increase in the confidence and reliability in the detection and quantification of metabolite NMR resonances.

## 3. Discussion

Metabolites are influenced both by the genome and the environment, and thus provide the most comprehensive readout for the state of an individual [36,37,38]. By monitoring changes in metabolites, it is possible to develop novel biomarkers that reveal important health information. Indeed, altered metabolite levels have been observed in many diseases, including diabetes [39], neurodegeneration [40], cancer [4], cardiovascular disease [6], and even aging [3]. Furthermore, in a series of separate studies, we have identified metabolite biomarkers of response (BoRs) that correlate with drug responsiveness for metastatic breast cancer patients treated with CDK4/6 inhibitors as well as the anti-HER2 therapy trastuzumab; and for gastrointestinal stromal tumor (GIST) patients treated with tyrosine kinase inhibitors [41,42,43]. While additional validation studies are required, these preliminary results suggest the exciting possibility that metabolite-based biomarkers have for designing optimal treatment strategies for individual patients, which is a major goal of precision medicine.

To uncover metabolite BoRs, it is first necessary to accurately measure metabolite levels in biospecimens collected from a large number of patients so that the relative metabolite concentration can be correlated with disease outcomes and/or a drug response. NMR and mass spectrometry (MS) are the two most commonly used analytical platforms for measuring metabolites. Traditionally, MS has been favored due to its high sensitivity, dynamic range, and potential for high throughput. There are numerous sensitive LC-MS methods reported in the literature for the identification of endogenous metabolites in human plasma [19,20,21,44,45,46]. For example, amino acids are routinely detected at submicromolar concentrations (0.01 to 0.04 µM) by these targeted LC-MS methods. In contrast, NMR-based approaches typically detect plasma concentrations in the micromolar (3–10 µM) range [44,45,46]. However, MS can suffer from reproducibility issues, requires chromatography because of the narrow molecular-weight distribution of metabolites, and still faces challenges in metabolite identification [9]. Conversely, NMR is highly reproducible and can reveal structural information to facilitate metabolite identification. However, NMR is limited by sensitivity and spectral overlap [47]. Multidimensional NMR can overcome some of these challenges but requires extremely long experimental times that are not practical for the large number of samples needed for BoR discovery. Efforts to increase the throughput of NMR are actively being explored. We and others have demonstrated that NUS can accelerate NMR acquisition times to meet the high-throughput demands of metabolomics [13,14,15]. In an approximate one-hour experiment, we verified that intensity measurements from NUS ^1^H-^13^C HSQC spectra are highly reproducible and can facilitate the detection of a wide variety of metabolites in the low micromolar range. 

In this study, we sought to explore additional techniques to extend the limit of metabolite detection by multidimensional NMR. As a first step, we assessed the effect of the d1 relaxation delay on S/N. The relaxation delay is the experimental time between scans in an NMR experiment to allow the nuclear spins to return to equilibrium, which is influenced by T_1_ longitudinal relaxation rates of each nuclei in the sample. For quantitative NMR, it is suggested to set d1 to at least 5 times the slowest T_1_ [17]. For metabolomics, this is not practical as T_1_s can be several seconds in length or longer. Instead, d1 is commonly set to a shorter, predetermined value for semi-quantification. Herein, we demonstrated that a decrease in d1 from 1.5 s to 0.8 s enabled an increase in the number of scans from 36 to 72, which led to an overall improvement in S/N and an increase in the mean signal intensity for metabolite resonances. Notably, this was accomplished without increasing the total time to acquire the NMR spectrum. Any further reduction in d1 was observed to result in severe signal artifacts from the solvent. 

With the optimal d1 selected, we next sought to manipulate the T_1_s of metabolite nuclei through PRE. PRE accelerates spin relaxation due to induced magnetic dipolar interactions with unpaired electrons. PRE-based applications have been used for macromolecular structure determination, characterizing long-range interactions and identifying transiently populated states of proteins and complexes [28,48,49]. PRE agents also provide the foundation for contrast agents in magnetic resonance imaging (MRI) [50]. Previous metabolomics studies have suggested that the addition of PRE-agents, Cu(EDTA) or Gd, can decrease T_1_ relaxation times for metabolites [13,17]. We observed similar trends using our standard 25% NUS ^1^H-^13^C HSQC experimental parameters and noted that the addition of Gd led to an overall improvement in S/N and mean signal intensity for metabolites in a model mixture and from plasma samples. Although the average increased fold change in intensity with Gd was relatively modest, we did observe a significant improvement (>2-fold increase) for metabolite resonances with the lowest signal intensity. For metabolomics studies, the ability to accurately detect and quantify a broad range of metabolites that span different chemical classes and concentration ranges is paramount. Thus, an increase in the NMR signal intensity for low abundant metabolites suggests that the addition of Gd could improve the coverage of the metabolome. Indeed, both the lower limit of detection and quantification (LoD/LoQ) were significantly improved in the presence of Gd. In our previous results, the LoD and LoQ for a model mixture of metabolites was 19.1 μM and 65.6 μM, respectively. For the same model mixture, the LoD and LoQ decreased by more than 2-fold to 7.8 μM and 26 μM, respectively, by decreasing the d1 and by the addition of Gd.

## 4. Materials and Methods

Commercially available analytical standards were used to prepare model mixtures of metabolites, Reference 1 and Reference 2 (Tables S1 and S2): acetylcholine chloride (C_7_H_15_NO_2_·HCl, >99%), L-arginine (C_6_H_14_N_4_O_2_, >98%), L-glutamine (C_5_H_10_N_2_O_3_, >99%), D-alpha-hydroxyglutaric acid disodium salt (C_5_H_6_Na_2_O_5_, >98%), α-ketoglutaric acid disodium salt dihydrate (C_5_H_4_Na_2_O_5_·2H_2_O, >98%), adenosine 5-monophosphate disodium (C_10_H_12_N_5_Na_2_O_7_P, >99%), D-(-)-fructose (C_6_H_12_O_6_, >99%), guanosine 5-triphosphate sodium salt (C_10_H_16_N_5_O_14_P_3_·xNa + yH_2_O, >95%), lithium potassium acetyl phosphate (C_2_H_3_KLiO_5_P, >97%), L-ornithine hydrochloride (C_5_H_12_N_2_O_2_·HCl, >98%), β-nicotinamide adenine dinucleotide hydrate (C_21_H_27_N_7_O_14_P_2_·xH_2_O, >98%), DL-malic acid (C_4_H_6_O_5_, >99%), D-ribose 5-phosphate disodium salt dihydrate (C_5_H_9_Na_2_O_8_P·2H_2_O, >99%), sodium succinate dibasic hexahydrate (C_4_H_4_Na_2_O_4_·6H_2_O, >99%), sodium acetate (C_2_H_3_NaO_2_, >99%), sodium L-lactate (C_3_H_5_NaO_3_, >99), sodium citrate tribasic dihydrate (C_6_H_5_O_7_Na_3_·2H_2_O, >99%), sodium fumarate dibasic (C_4_H_2_Na_2_O_4_, >98%), sodium pyruvate (C_3_H_3_NaO_3_, >99%), uridine 5-diphosphate (C_9_H_12_N_2_Na_2_O_12_P_2_·xH_2_O, >96%), L-alanine (C_3_H_7_NO_2_, >98%), L-cysteine (C_3_H_7_NO_2_S, >98%), D-(+)-glucosamine hydrochloride (C_6_H_13_NO_5_·HCl, >99%), D-(+)-glucose (C_6_H_12_O_6_, >99.5%), choline chloride (C_5_H_13_NO·HCl, >99%), cytidine (C_9_H_13_N_3_O_5_, >99%), L-leucine (C_6_H_13_NO_2_, >98.5%), L-glutamic acid monosodium salt monohydrate (C_5_H_8_NNaO_4_·H_2_O, >99%), L-histidine (C_6_H_11_N_3_O_3_·HCl, >98.5%), L-lysine, monohydrochloride (C_6_H_14_N_2_O_2_·HCl, >98.5%). All the compounds were obtained from Sigma-Aldrich. Deuterium oxide (D_2_O, 99.0%) was purchased from Cambridge Isotope Laboratory, Inc., Andover, MA. Pooled human plasma (apheresisderived, K2EDTA) was purchased from innovative research, Novi, MI. Paramagnetic relaxation agents gadobutrol (Gd) (C_18_H_31_GdN_4_O_9_, >99.9%) and copper (II) disodium ethylenediaminetetraacetate tetrahydrate (Cu- EDTA) (C_10_H_12_CuN_2_Na_2_O_8_·4H_2_O) were procured from MedChemExpress, Monmouth Junction, NJ and TCI America, Portland, OR respectively. The NMR reference standard, deuterated 3-(trimethylsilyl)-1-propanesulfonic acid sodium salt (DSS-d6, 98%) was purchased from Cambridge Isotope Laboratory, Andover, MA, USA.

### 4.1. NMR Sample Preparation

Reference 1 and Reference 2 were prepared as previously described [15]. Human plasma extraction: metabolites were extracted from 1 mL of human plasma via a methanol and chloroform liquid–liquid extraction. The aqueous phase was transferred to a 15 mL Falcon tube and freeze-dried. The powder was reconstituted in 180 µL of 50 mM phosphate buffer at pH 7.4 in D_2_O, and then immediately transferred to a 3 mm NMR tube for NMR data collection. The NMR standard, DSS-d6, was added to each sample for chemical shift referencing.

### 4.2. NMR Experiments and Data Processing

All NMR spectra were acquired on a Bruker AVANCE III solution-state NMR spectrometer equipped with a liquid helium-cooled TCI (H/F, C, N), deuterium lock, and a cryoprobe operating at a frequency of 599.773010 MHz for proton and 150.822998 MHz for carbon. NUS schedules were generated using a Poisson gap distribution with a sinusoidal weight of two and random seed generator [51]. The same 25% NUS schedule and seed were used for all experiments. All NMR data were collected at 298 K.

The spectral widths along the direct and the indirect dimensions were set at 9578.544 and 24,132.982 Hz, respectively. The number of complex points in the direct dimension was set at 512, and in the indirect dimension set at 32 with a 25% NUS sampling schedule. The number of scans for the 1D ^1^H experiments was set to 64 (d1 = 1.5 s), 72 (d1 = 1.2 s), 84 (d1 = 0.8 s), and 92 (d1 = 0.6 s). The number of scans for the 2D ^1^H-^13^C HSQC experiments was set to 36 (d1 = 1.5 s), 48 (d1 = 1.2 s), 72 (d1 = 0.8 s) and 84 (d1 = 0.6 s), respectively. The scan numbers were selected such that the total acquisition time for each 1D- and 2D- experiment was on average 246 s, and 69 min., respectively. The transmitter frequency offset was set to 75 ppm in the ^13^C dimension and 4.7 ppm in the ^1^H dimension. 

The spectral data were processed using the NMRPipe software package, as previously described [52]. The NUS data were reconstructed using iterative soft thresholding according to the hmsIST algorithm [51] to generate the same number of direct dimension data points and twice the number of indirect dimension data points, 512 (N_2_) × 256 (N_1_). Both the NUS and US NMR data were zero-filled, Fourier-transformed and manually phase-corrected to yield a final digital resolution of 2048 (N_2_) × 2048 (N_1_) points. Chemical shift queries, metabolite identifications and quantifications were performed using the COLMARm NMR webserver (http://spin.ccic.ohio-state.edu/index.php/colmar (accessed on 04/02/21) [35]. The metabolite list is presented in the Supplementary Materials section for Reference 1 (Table S1) and Reference 2 (Table S2). The resonance assignments were used as previously reported [15].

## 5. Conclusions

In this study, we demonstrated the ability of Gd to improve the sensitivity of 2D NUS NMR spectra for the analysis of metabolomics samples. The addition of Gd led to an overall improvement in S/N and mean signal intensity for metabolites in both a model mixture and plasma samples. In the model mixture, the addition of Gd led to a 1.25-fold improvement in NMR signal intensities, which resulted in 1.7- and 1.6-fold improvements in LOD and LOQ, respectively. Interestingly, a significant improvement (>2-fold increase) was observed for metabolites with the lowest peak intensities, which suggests that the combination of Gd with NUS may improve the coverage of the plasma metabolome. The addition of Gd also maintained the highly linear intensity measurements that were correlated with a wide range of metabolite concentrations (50 μM to 2 mM). The reproducibility of intensity measurements, as noted by a decrease in %CV for both the model mixture (8% to 7%) and the plasma samples (15% to 10%), was similarly improved with the addition of Gd. Collectively, our results suggest that supplementing metabolomics samples with 0.25 mM Gd can improve the sensitivity of 2D NUS ^1^H-^13^C HSQC spectra and enhance the overall quality of the resulting data analysis. The routine adoption of PRE by the metabolomics community may expand the utility of multidimensional NMR to empower future biomarker discoveries.

## Figures and Tables

**Table 1:** Reference 2 metabolite resonances and their correlation coefficient (R^2^) for each resonance.

Metabolites/R^2^	1	2	3	4	5	6	7	8	9	10	11	12	13
NAD	0.996	0.999	0.999	0.997	1.000	0.999	0.998	0.999	1.000	0.999	1.000	0.999	0.999
NADPH	1.000	1.000	0.886	0.929	0.995	0.966	0.967	0.966	0.972	0.968	0.950		
Cytidine	0.912	0.999	0.997	0.998	0.997	0.998	0.998						
UDP	0.999	0.999	0.999	0.999	0.999	0.999	1.000						
Fructose	1.000	1.000	1.000	1.000	0.984	0.996							
AMP	0.951	0.973	0.970	0.992	0.901	0.994							
Lysine	0.998	0.999	0.999	0.998	0.942								
Histidine	0.998	1.000	0.998	0.999	0.998								
Glucose	0.995	0.997	0.993	0.996	0.974								
Ribose 5P	0.983	0.990	0.989	0.996	0.994								
Glucosamine	0.997	0.959	0.992	0.951									
2-HG	0.994	0.998	0.999	0.984									
Leucine	0.998	0.997	0.999	0.997									
Nicotinic acid	0.998	0.996	0.994	0.997									
Acetylcholine	0.999	0.989	0.998	0.998									
Glutamic acid	0.998	0.998	0.952										
Malic acid	0.993	0.989	0.996										
Arginine	0.991	0.997	0.999										
Ornithine	0.998	0.996	1.000										
Choline	0.999	0.997	0.996										
Glutamine	0.998	0.998	0.998										
GTP	0.998	0.998	0.967										
Citrate	0.990	0.998											
Alanine	0.998	0.995											
Lactic acid	0.999	0.997											
Pyruvic acid	0.998												
Acetic acid	0.990												
Fumaric acid	0.989												
Succinic acid	0.998												

**Table 2:** Limit of detection (LOD) for measured metabolite resonances.

	LOD (µM) per NMR Resonance													
Metabolites	1	2	3	4	5	6	7	8	9	10	11	12	13	Minimal Conc. (µM)
NAD	12.7 ± 0.49	12.58 ± 0.26	26.05 ± 0.32	12.2 ± 0.04	10.61 ± 0.19	15.54 ± 0.97	7.9 ± 0.08	13.48 ± 0.15	16.5 ± 0.04	14.41 ± 0.11	12.98 ± 0.16	9.2 ± 0.09	12.9 ± 0.02	7.90 ± 0.08
NADPH	26.85 ± 0.21	21.76 ± 0.99	7.46 ± 0.17	19.01 ± 0.38	13.4 ± 0.48	10.66 ± 0.42	14.4 ± 0.14	25.37 ± 0.5	19.46 ± 0.96	12.98 ± 0.27	24.4 ± 0.62			7.46 ± 0.17
Cytidine	18.1 ± 0.66	8.44 ± 0.02	9.04 ± 0.08	7.4 ± 0.00	6.62 ± 0.05	7.86 ± 0.06	7.89 ± 0.05							6.62 ± 0.05
UDP	6.78 ± 0.09	9.47 ± 0.15	9.82 ± 0.2	9.85 ± 0.01	8.69 ± 0.11	10.65 ± 0.15	10.69 ± 0.05							6.78 ± 0.09
Fructose	19.58 ± 0.93	13.82 ± 0.45	13.85 ± 0.44	18.89 ± 1.17	18.71 ± 0.33	9.49 ± 0.09								9.49 ± 0.09
AMP	8.35 ± 1.68	12.14 ± 1.54	3.94 ± 0.16	13.17 ± 2.97	10.75 ± 1.7	14.96 ± 2.96								3.94 ± 0.16
Lysine	23.24 ± 0.19	10.07 ± 0.24	9.28 ± 0.13	6.59 ± 0.03	3.6 ± 0.1									3.60 ± 0.10
Histidine	19.15 ± 2.19	18.79 ± 1.91	10.85 ± 1.66	7.9 ± 0.38	11.3 ± 2.09									7.90 ± 0.38
Glucose	21.62 ± 3.18	18.61 ± 2.01	16.35 ± 1.1	14.32 ± 0.11	15.79 ± 2.56									14.32 ± 0.11
Ribose 5P	41.21 ± 2.58	30.31 ± 1.82	21.22 ± 0.59	8.98 ± 0.26	19.94 ± 0.71									8.98 ± 0.26
Glucosamine	30.31 ± 1.11	19.2 ± 0.570.57	33.83 ± 1.51	22.32 ± 1.71										19.20 ± 0.57
2-HG	33.83 ± 1.76	33.3 ± 1.89	25.95 ± 0.75	12.7 ± 0.3										12.70 ± 0.3
Leucine	5.64 ± 0.98	5.58 ± 1.07	20.77 ± 1.54	11.49 ± 1.85										5.58 ± 1.07
Nicotinic acid	10.78 ± 0.03	9.71 ± 0.19	8.04 ± 0.16	7.26 ± 0.01										7.26 ± 0.01
Acetylcholine	8.56 ± 0.12	0.71 ± 0.02	8.42 ± 0.1	8.06 ± 0.07										0.71 ± 0.02
Glutamic acid	22.1± 1.1	8.87 ± 0.13	4.09 ± 0.08											4.09 ± 0.08
Malic acid	30.52 ± 2.05	30.12 ± 0.41	8.98 ± 0.26											8.98 ± 0.26
Arginine	25.49 ± 2.75	9.06 ± 0.09	6.09 ± 0.06											6.09 ± 0.06
Ornithine	28.27 ± 1.77	4.59 ± 0.05	6.71 ± 0.05											4.59 ± 0.05
Choline	1.41 ± 0.05	8.09 ± 0.3	7.76 ± 0.29											1.41 ± 0.05
Glutamine	8.32 ± 0.13	9.46 ± 0.22	5.06 ± 0.07											5.06 ± 0.07
GTP	11.66 ± 0.18	15.58 ± 0.51	7.54 ± 1.44											7.54 ± 1.44
Citrate	8.2 ± 1.11	8.15 ± 1.21												5.14 ± 0.01
Alanine	5.14 ± 0.01	13.4 ± 0.08												8.15 ± 1.21
Lactic acid	5.4 ± 0.02	13.89 ± 0.22												5.4 ± 0.02
Pyruvic acid	30.79± 2.83													30.79 ± 2.83
Acetic acid	7.21 ± 0.08													7.21 ± 0.08
Fumaric acid	5.6 ± 0.14													5.67 ± 0.12
Succinic acid	3.21 ± 0.03													3.21 ± 0.03

**Table 3:** Limit of quantification (LOQ) for measured metabolite resonances.

	LOQ (μM) per NMR Resonance													
Metabolites	1	2	3	4	5	6	7	8	9	10	11	12	13	Minimal Conc. (µM)
NAD	42.34 ± 1.62	41.92 ± 0.88	86.83 ± 1.06	40.66 ± 0.13	35.37 ± 0.62	51.78 ± 3.22	26.35 ± 0.27	44.92 ± 0.51	55.01 ± 0.14	48.03 ± 0.38	43.26 ± 0.55	30.66 ± 0.3	43.01 ± 0.06	26.35 ± 0.27
NADPH	89.49 ± 0.7	72.53 ± 3.29	24.87 ± 0.55	63.38 ± 1.25	44.66 ± 1.61	35.52 ± 1.39	48.01 ± 0.47	84.56 ± 1.67	64.88 ± 3.19	43.27 ± 0.91	81.33 ± 2.07			35.52 ± 1.39
Cytidine	60.34 ± 2.2	28.12 ± 0.08	30.15 ± 0.28	24.68 ± 0.01	22.05 ± 0.18	26.2 ± 0.19	26.29 ± 0.15							22.05 ± 0.18
UDP	22.59 ± 0.29	31.55 ±0.51	32.74 ± 0.68	32.85 ± 0.03	28.95 ± 0.36	35.49 ± 0.49	35.64 ± 0.15							22.59 ± 0.29
Fructose	65.27 ± 3.1	46.06 ±1.5	46.18 ± 1.47	62.98 ± 3.89	62.38 ± 1.09	31.63 ± 0.29								31.63 ± 0.29
AMP	27.83 ± 5.61	40.46 ± 5.12	13.13 ± 0.52	43.92 ± 9.89	35.84 ± 5.68	49.86 ± 9.88								13.13 ± 0.52
Lysine	77.47 ± 0.64	33.57 ± 0.8	30.92 ± 0.44	21.95 ± 0.08	12.01 ± 0.34									12.01 ± 0.34
Histidine	63.83 ± 7.29	62.63 ± 6.38	36.18 ± 5.53	26.32 ± 1.25	37.67 ± 6.97									26.32 ± 1.25
Glucose	72.07 ±10.59	62.02 ± 6.69	54.5 ± 3.66	47.74 ± 0.36	52.63 ± 8.54									47.74 ± 0.36
Ribose 5P	137.37 ± 8.59	101.02 ± 6.08	70.73 ± 1.97	30.11 ± 0.89	66.45 ± 2.38									30.11 ± 0.89
Glucosamine	101.02 ± 3.71	42.87 ± 2.83	112.76 ± 5.02	74.4 ± 5.69										42.87 ± 2.83
2-HG	112.76 ± 5.86	110.99 ± 6.3	86.49 ± 2.51	42.34 ±1.00										42.34 ± 1.00
Leucine	18.78 ± 3.27	18.61 ± 3.58	69.24 ± 5.13	38.28 ± 6.16										18.61 ± 3.58
Nicotinic acid	35.93 ± 0.1	32.36 ± 0.62	26.8 ± 0.54	24.19 ± 0.04										24.19 ± 0.04
Acetylcholine	28.54 ± 0.4	2.38 ± 0.08	28.08 + BL86 ± 0.33	26.88 ± 0.24										2.38 ± 0.08
Glutamic acid	73.67 ± 3.65	29.55 ± 0.44	13.64 ± 0.28											13.64 ± 0.28
Malic acid	101.74 ± 6.84	100.41 ± 1.36	29.92 ± 0.88											29.92 ± 0.88
Arginine	84.96 ± 9.16	30.2 ± 0.3	20.32 ± 0.19											20.32 ± 0.19
Ornithine	94.24 ± 5.91	15.3 ± 0.16	22.35 ± 0.18											15.30 ± 0.16
Choline	4.69 ± 0.15	26.98 ± 0.99	25.85 ± 0.97											4.69 ± 0.15
Glutamine	27.72 ± 0.44	31.54 ± 0.73	16.85 ± 0.24											16.85 ± 0.24
GTP	38.87 ± 0.6	51.94 ± 1.7	25.13 ± 4.8											25.13 ± 4.80
Citrate	27.33 ± 3.71	27.18 ± 4.03												27.18 ± 4.03
Alanine	17.15 ± 0.02	44.67 ± 0.26												17.15 ± 0.02
Lactic acid	18.08 ± 0.19	46.29 ± 0.74												18.08 ± 0.19
Pyruvic acid	102.65 ± 9.42													102.65 ± 9.42
Acetic acid	24.61 ± 0.35													24.61 ± 0.35
Fumaric acid	18.89 ± 0.39													18.89 ± 0.39
Succinic acid	10.69 ± 0.11													10.69 ± 0.11

## Data Availability

Data available on request due to proprietary restrictions. The data presented in this study are available on request from the corresponding author.

## Supplementary Materials

The following are available online. Figure S1. Chemical structure of Gadobutrol (Gd), Figure S2. Effect of addition of relaxation agents on 1H-13C HSQC 1D ^1^H spectral intensity for a model mixture (Reference 1) of metabolites in time equivalent experiments, Figure S3. Plots showing (a) mean peak intensity and (b) mean S/N for Reference 1 with and without addition of Gadobutrol, Figure S4. Linear regression curve of Reference 2 metabolites, Table S1. The list of metabolites in Reference 1 model mixture, Table S2. The list of metabolites in Reference 2 model mixture, Table S3. Comparison of LOD and LOQ at 0.8 D1 and at 1.5 D1 with and without Gd.

## Sample Availability

Samples are or not available from the authors.
