# Supplementary material for: Gadolinium-Based Paramagnetic Relaxation Enhancement Agent Enhances Sensitivity for NUS Multidimensional NMR-Based Metabolomics
Source: Molecules. 2021 Aug 24;26(17):5115. doi: 10.3390/molecules26175115 (PMC8433644; doi:10.3390/molecules26175115)
Supplement: Supplementary file 1 [file molecules-26-05115-s001.zip › molecules-1329608-SI.pdf]

Supplementary Figures

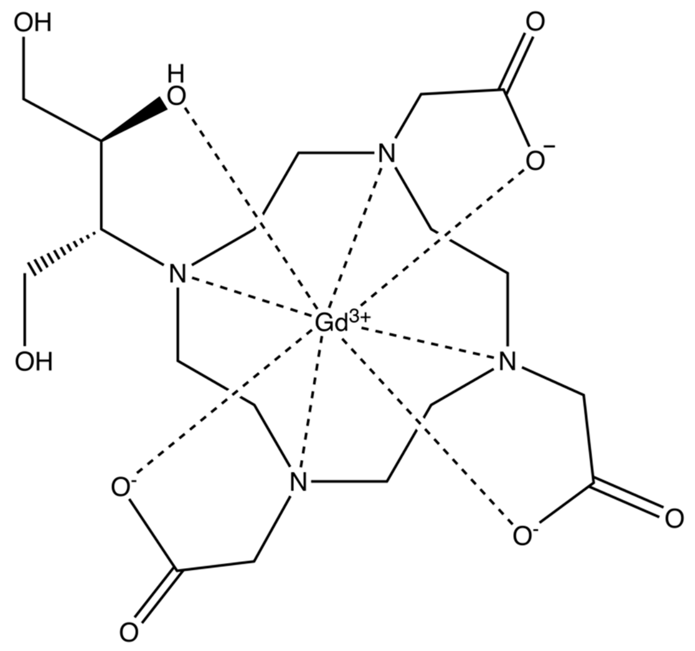

Figure S1. Chemical structure of Gadobutrol (Gd)

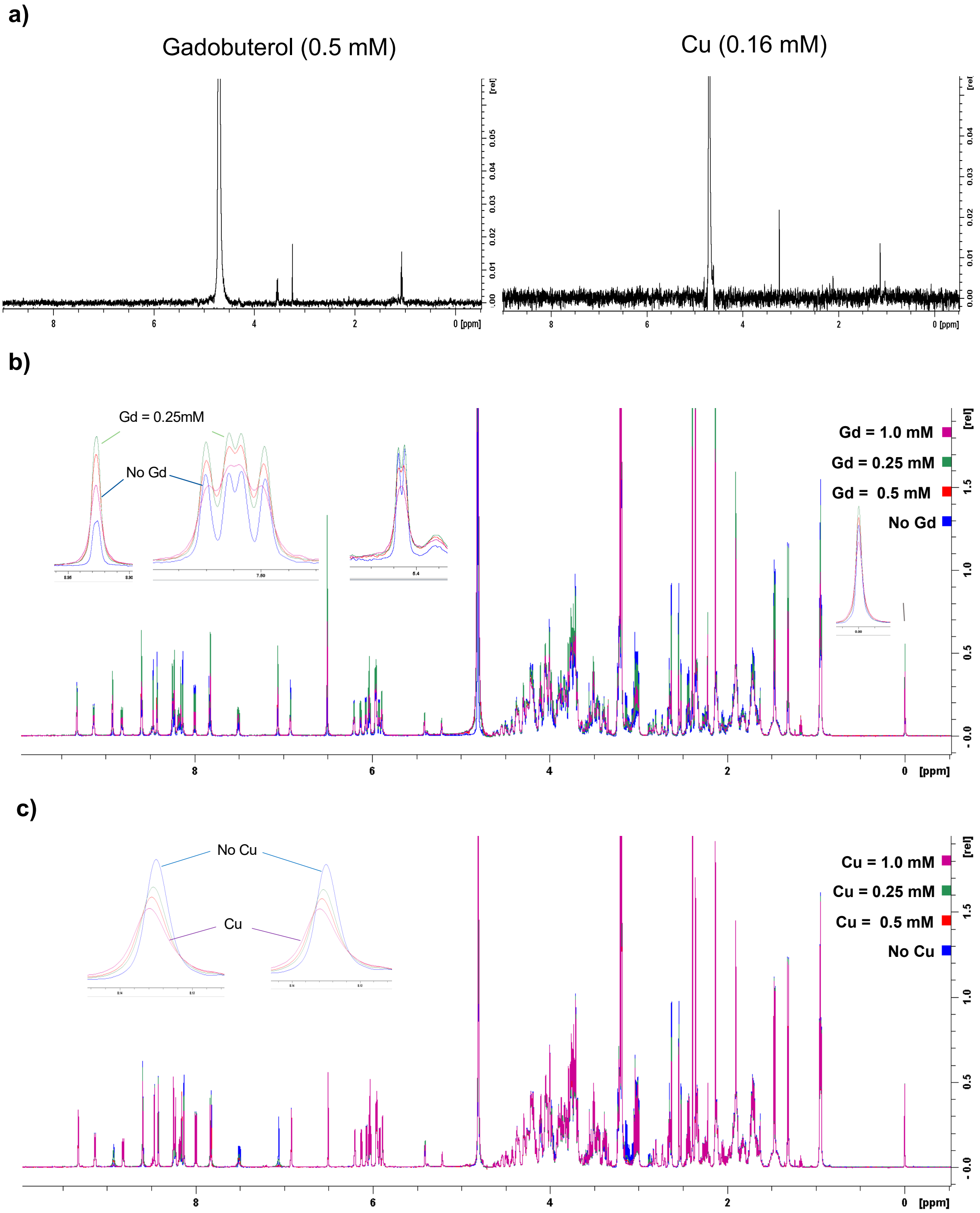

Figure S2. Effect of addition of relaxation agents on 1D ^{1}H spectral intensity for a model mixture (Reference 1) of metabolites in time equivalent experiments, **a)** 1D spectra of Gadobutrol (Gd) (left) and Copper-EDTA (Cu) (right), **b)** 1D overlay of reference 1 at different Gd concentrations, c) 1D overlay of reference 1 at different Cu-EDTA

a)

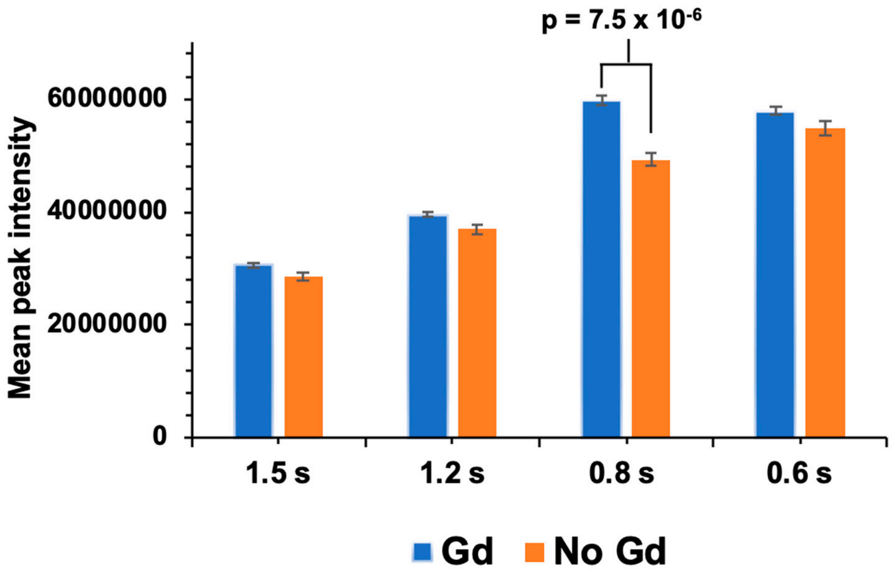

b)

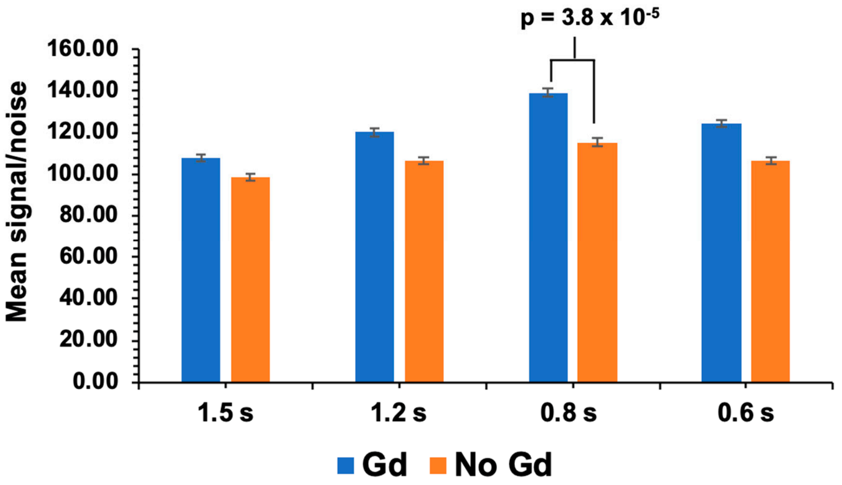

Figure S3. Plots showing (a) mean peak intensity and (b) mean S/N for Reference 1 with (blue) and without (orange) addition of Gadobutrol.

1. Glucose

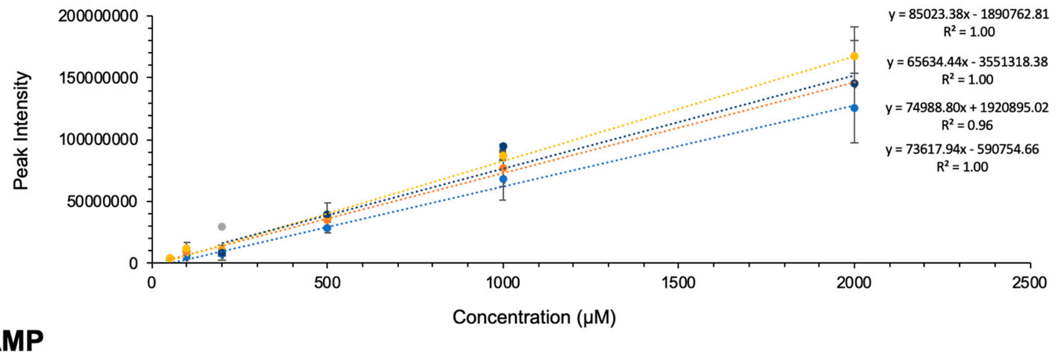

2. AMP

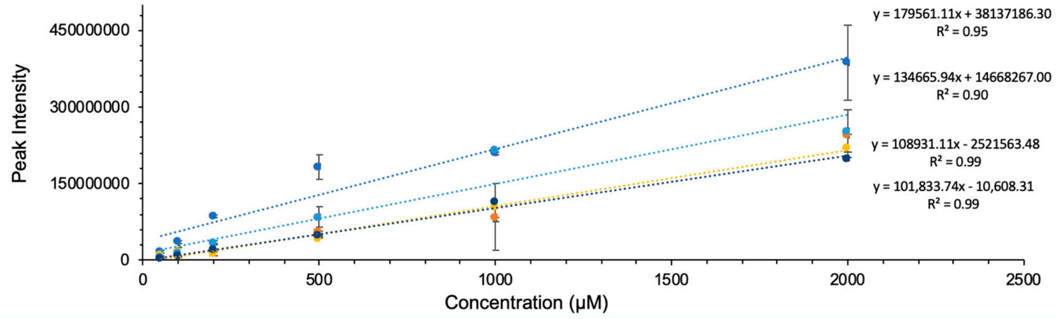

3. Alanine

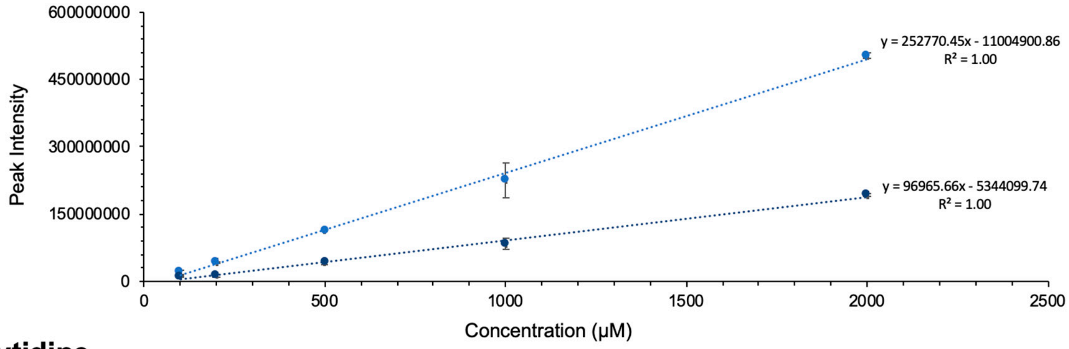

4. Cytidine

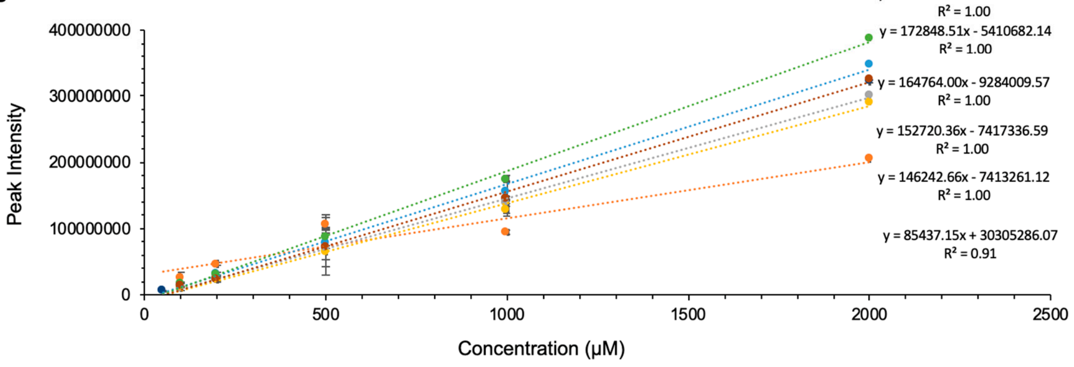

Figure S4. Linear regression curve of Reference 2 metabolites

5. Pyruvic acid

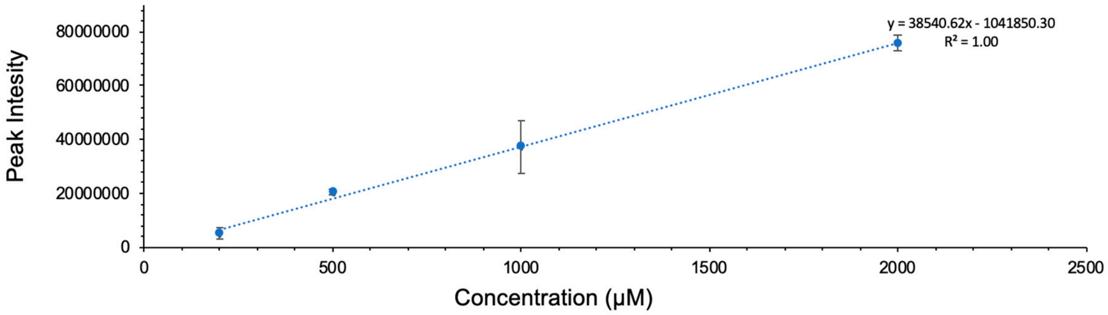

6. Glutamic acid

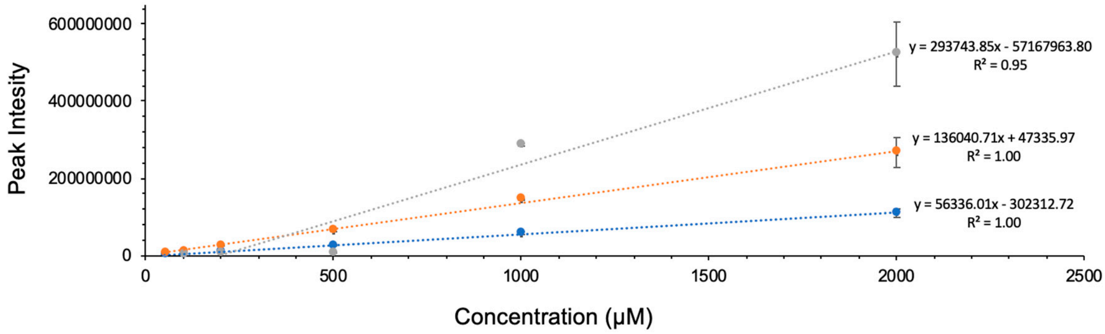

7. Succinic acid

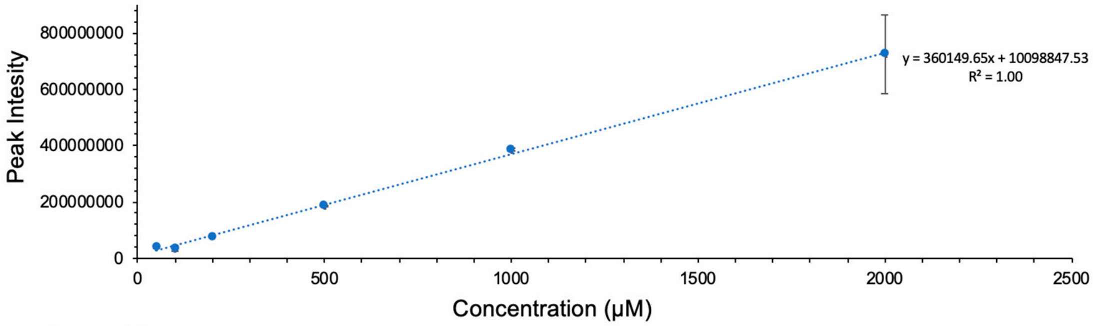

8. Fumaric acid

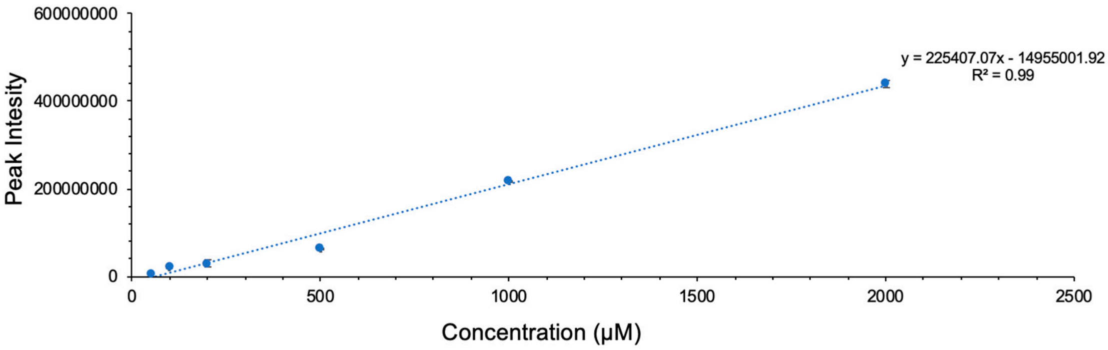

Figure S4. Linear regression curve of Reference 2 metabolites

9. Acetic acid

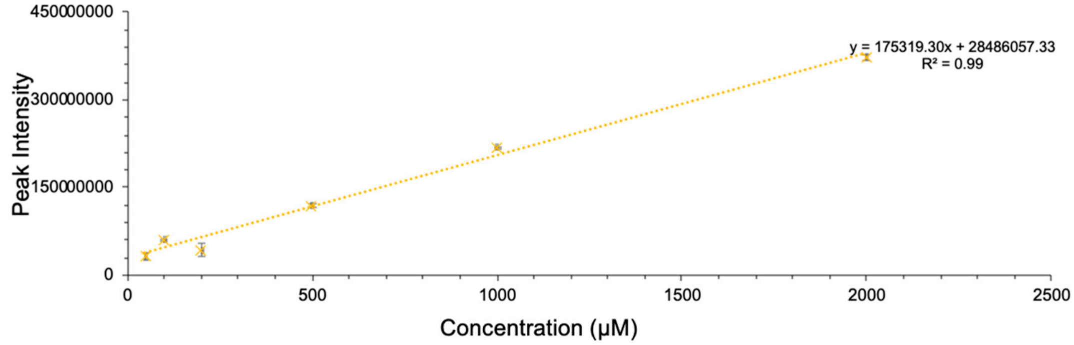

10. Citric acid

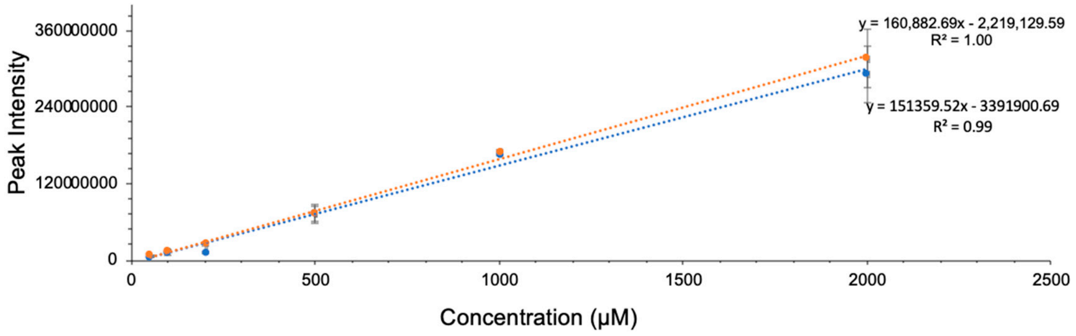

11. Maleic acid

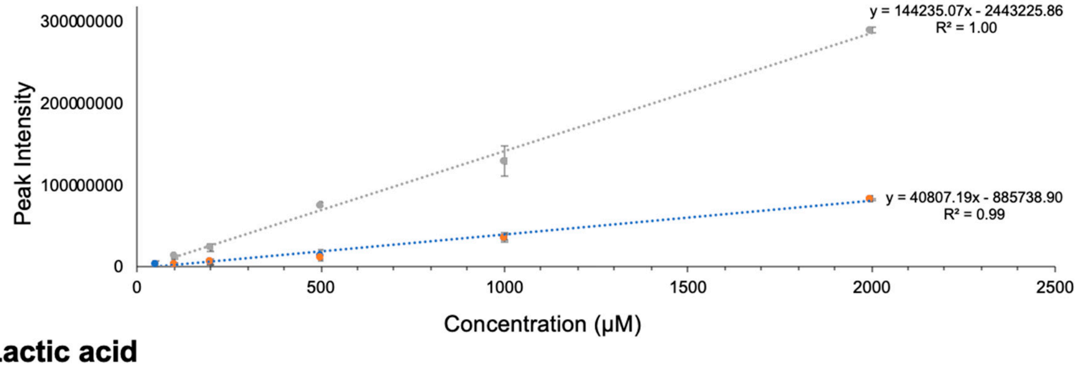

12. Lactic acid

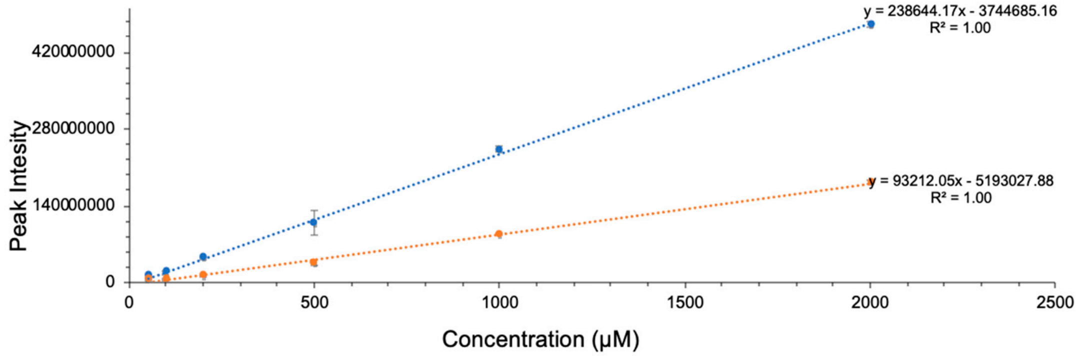

Figure S4. Linear regression curve of Reference 2 metabolites

13. Hydroxy Glutaric acid

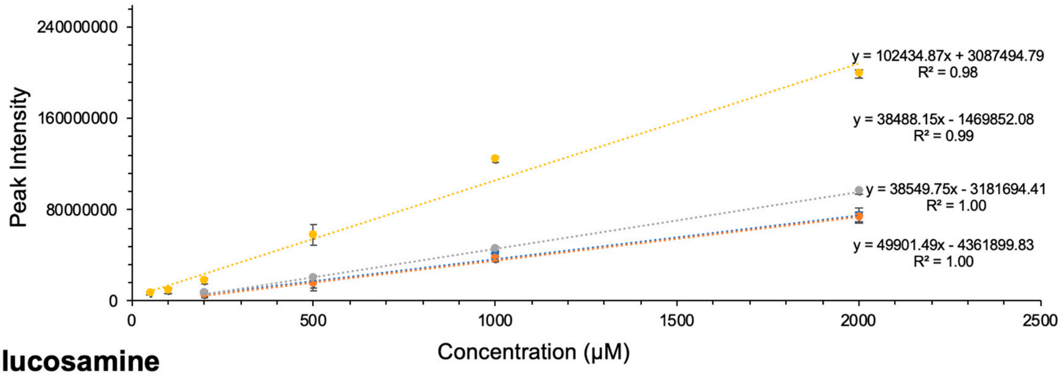

14. Glucosamine

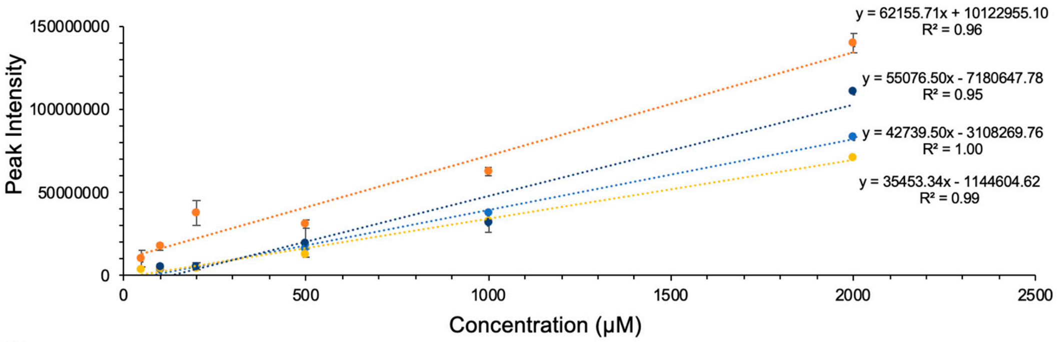

15. UDP

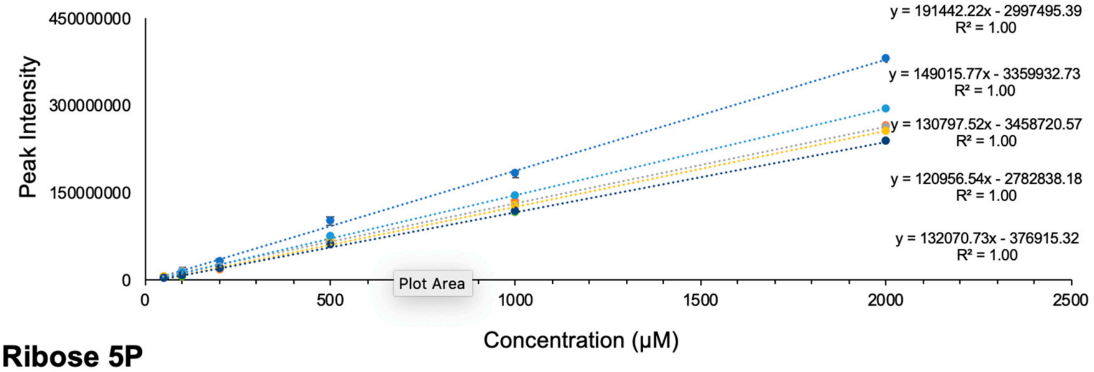

16. Ribose 5P

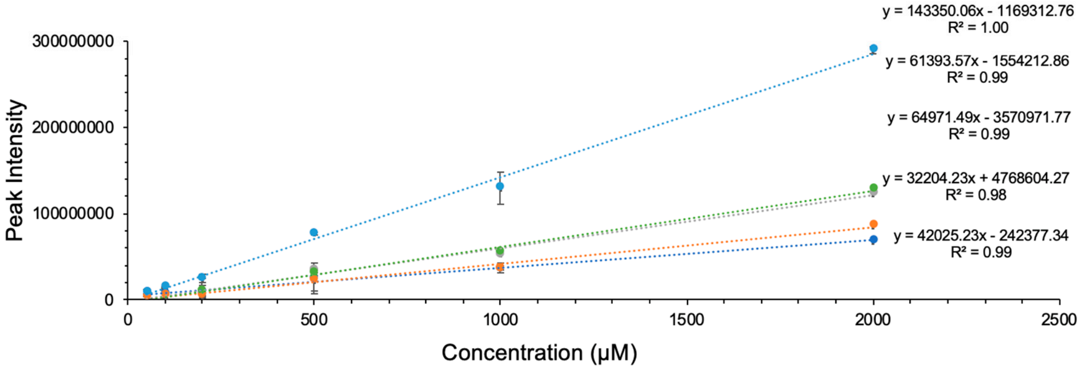

Figure S4. Linear regression curve of Reference 2 metabolites

17. Ornithine

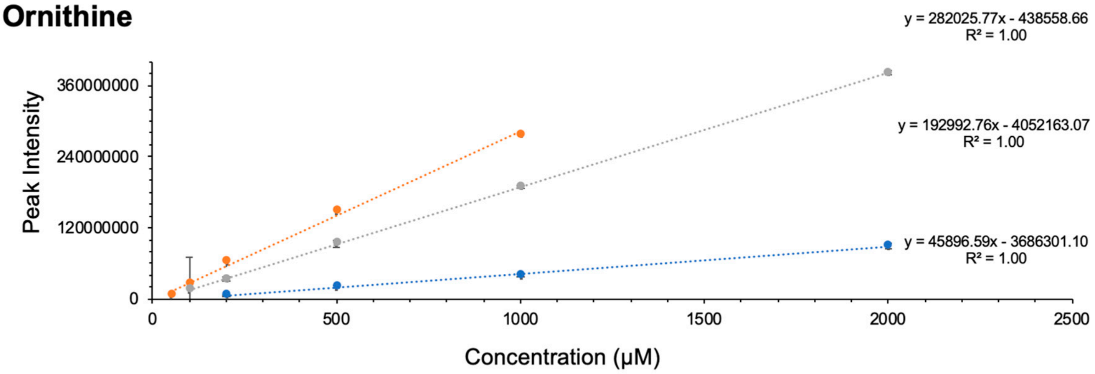

18. Glutamine

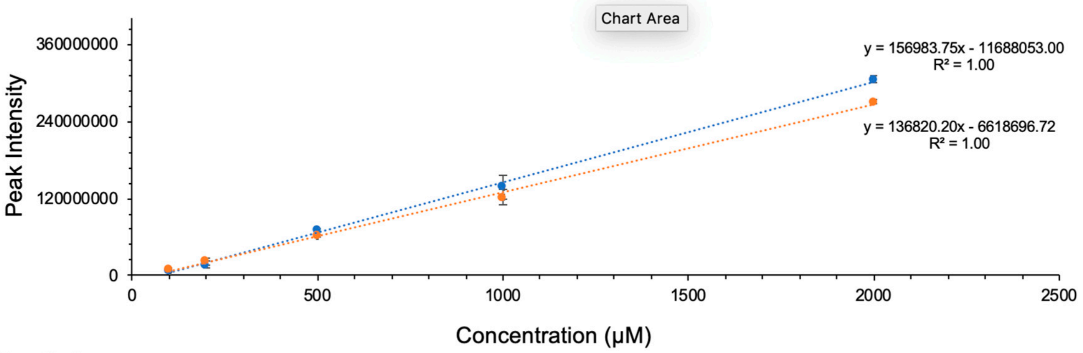

19. Arginine

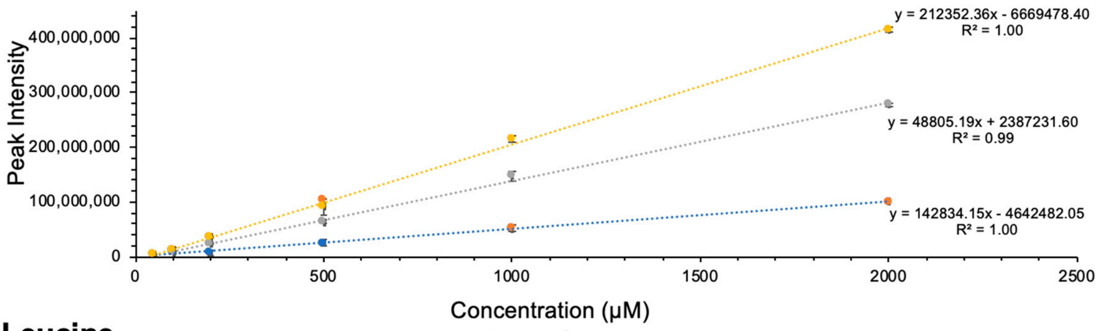

20. Leucine

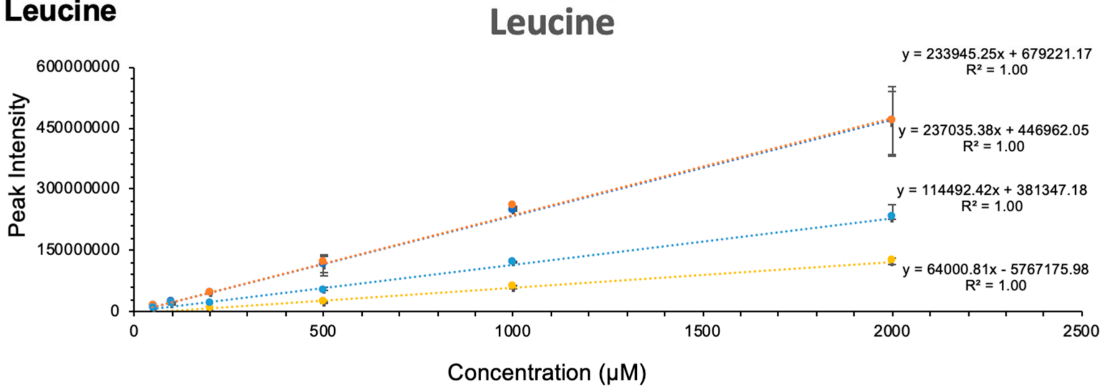

Figure S4. Linear regression curve of Reference 2 metabolites

21. Histidine

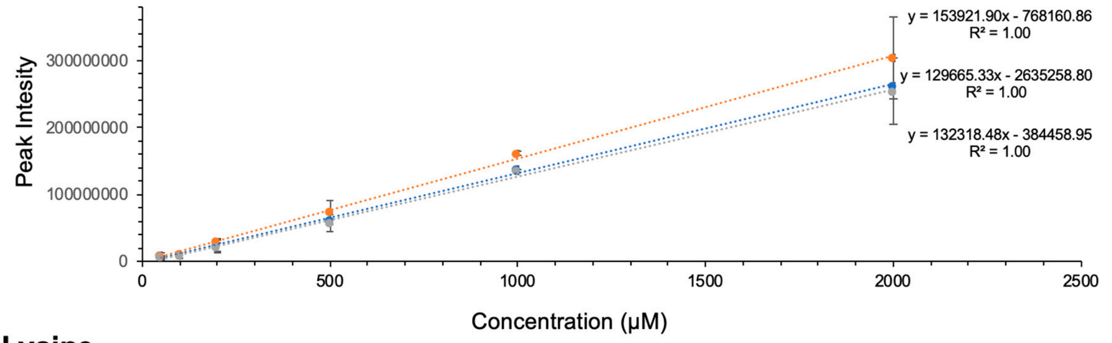

22. Lysine

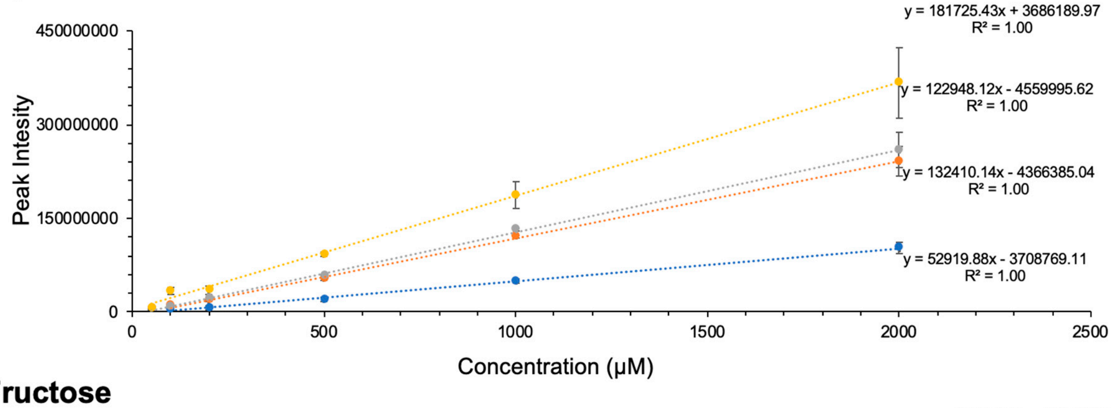

23. Fructose

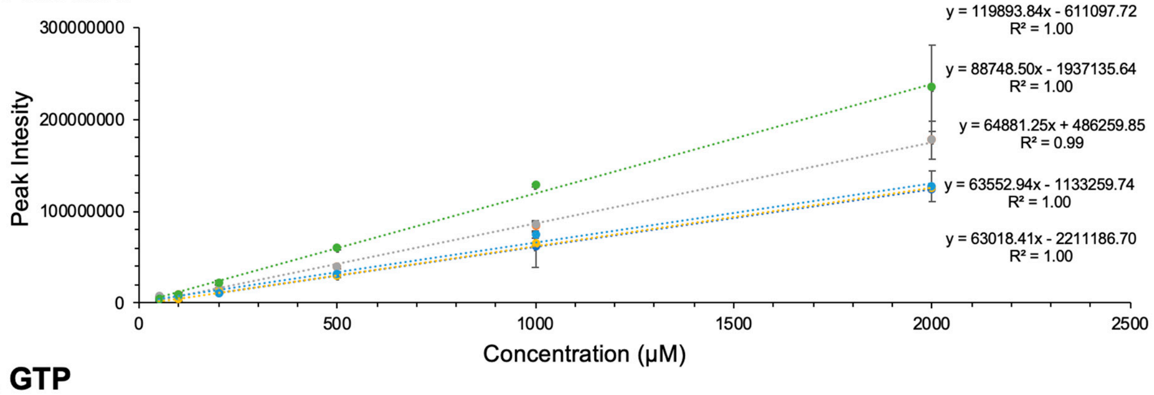

24. GTP

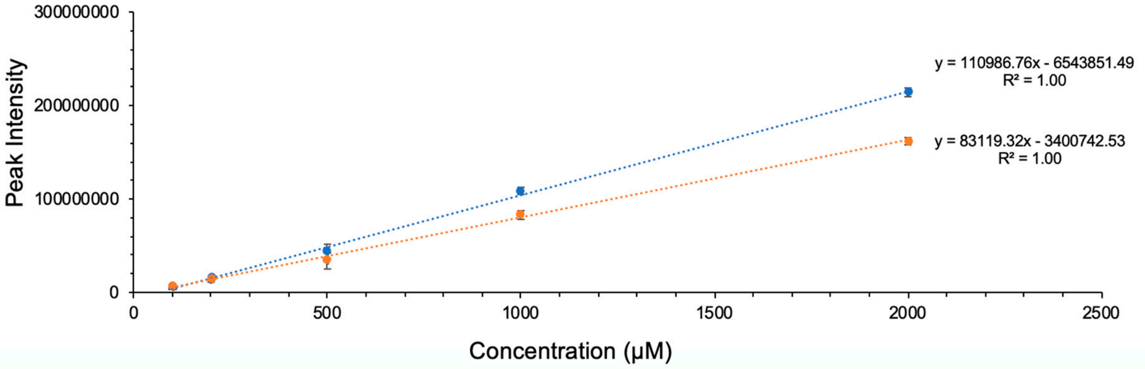

Figure S4. Linear regression curve of Reference 2 metabolites

25. Choline

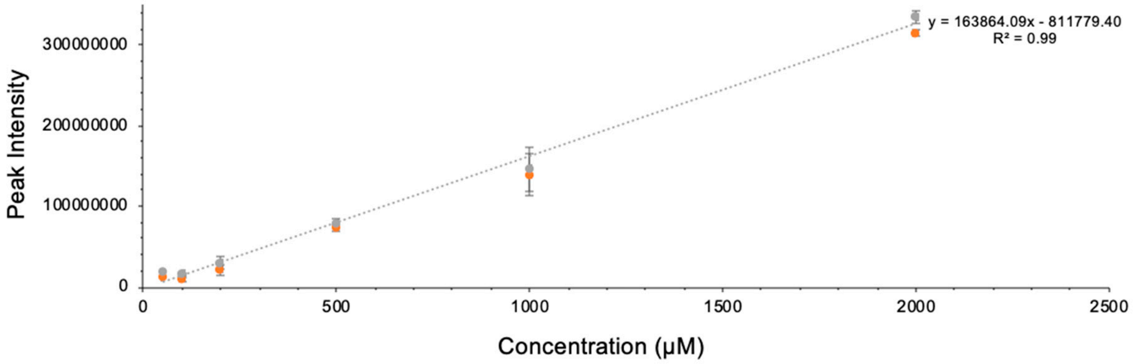

26. Acetyl choline

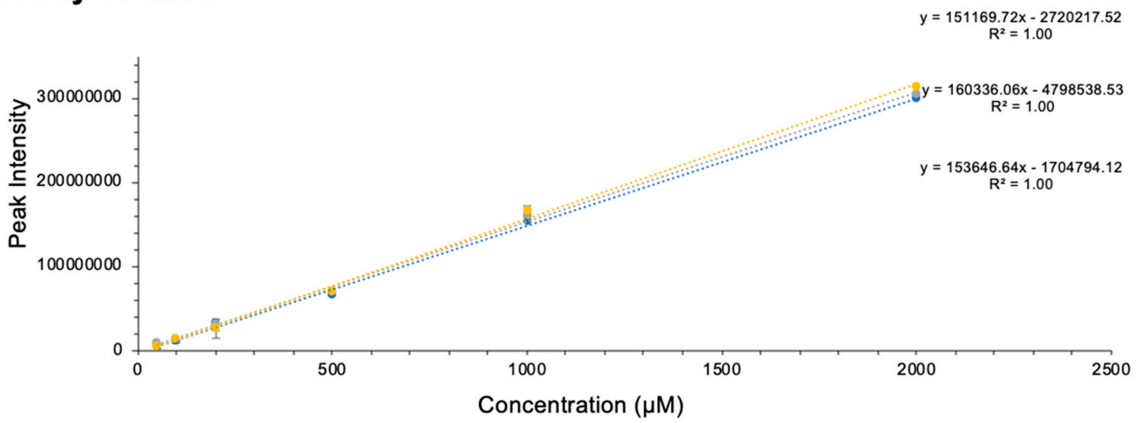

27. Nicotinic acid

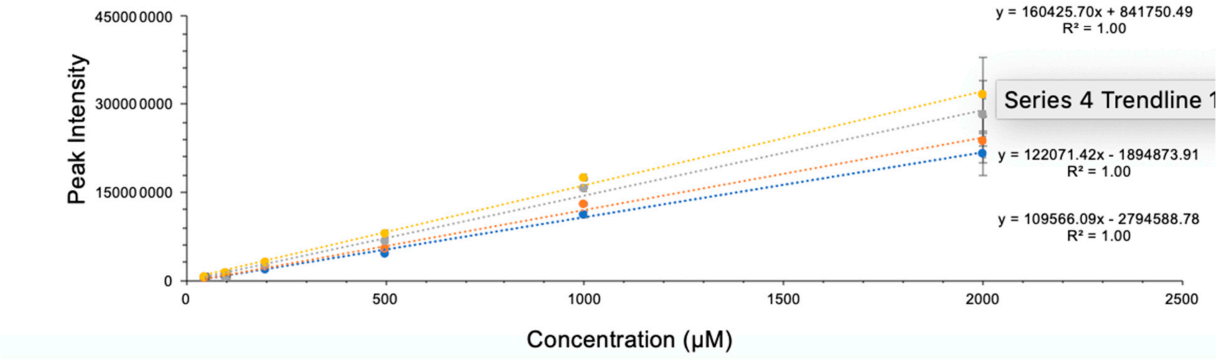

Figure S4. Linear regression curve of Reference 2 metabolites

28. NADPH

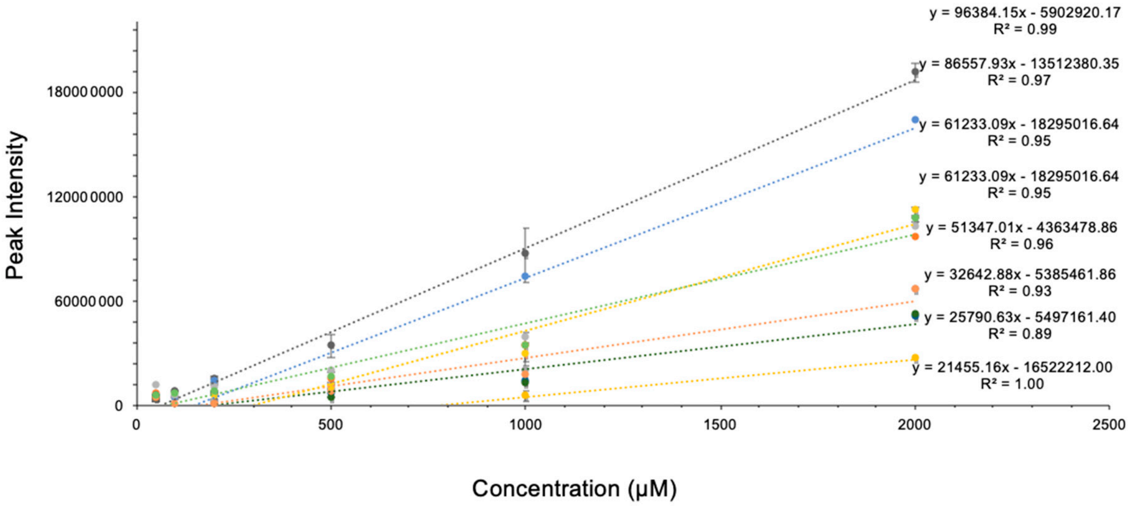

29. NAD

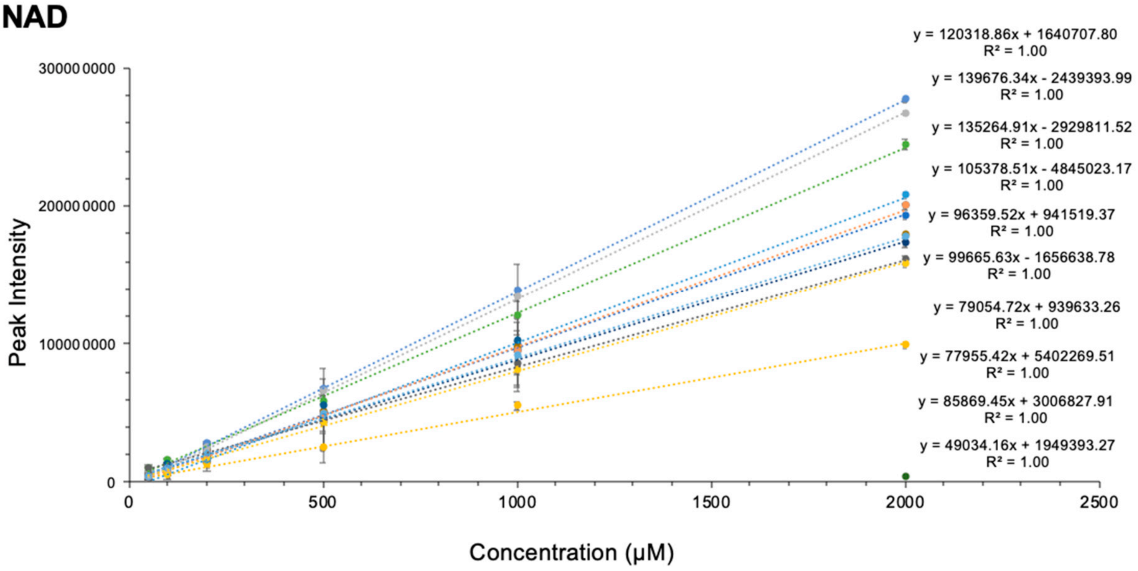

Figure S4. Linear regression curve of Reference 2 metabolites

Table S1. The list of metabolites in Reference 1 model mixture.

No.	Metabolites	Reference 1 Conc. (μM)
1	Glutamic acid	500
2	Acetylcholine	500
3	Leucine	500
4	Alanine	500
5	Lysine	500
6	Ornithine	500
7	Arginine	500
8	Choline	500
9	Glutamine	500
10	Nicotinic acid	500
11	Succinic acid	500
12	Acetic acid	500
13	Lactic acid	500
14	Malic acid	500
15	Citric acid	500
16	Pyruvic acid	500
17	NADPH	500
18	Fumaric acid	500
19	2-HG	500
20	Glucose	500
21	AMP	500
22	NAD	500
23	Glucosamine	500
24	UDP	500
25	GTP	500
26	Ribose 5-phosphate	500
27	Histidine	500
28	Cytidine	500
29	Fructose	500

Table S2. The list of metabolites in Reference 2 model mixture.

No.	Metabolites/Conc. (μM)	Std 1	Std 2	Std 3	Std 4	Std 5	Std 6
1	Glutamic acid	2000	50	100	200	500	1000
2	Acetylcholine	100	200	500	1000	2000	50
3	Leucine	500	1000	2000	50	100	200
4	Alanine	1000	2000	50	100	200	500
5	Lysine	2000	50	100	200	500	1000
6	Ornithine	50	100	200	500	1000	2000
7	Arginine	100	200	500	1000	2000	50
8	Choline	200	500	1000	2000	50	100
9	Glutamine	200	500	1000	2000	50	100
10	Nicotinic acid	1000	2000	50	100	200	500
11	Succinic acid	2000	50	100	200	500	1000
12	Acetic acid	50	100	200	500	1000	2000
13	Lactic acid	100	200	500	1000	2000	50
14	Malic acid	200	500	1000	2000	50	100
15	Citric acid	500	1000	2000	50	100	200
16	Pyruvic acid	1000	2000	50	100	200	500
17	NADPH	2000	50	100	200	500	1000
18	Fumaric acid	50	100	200	500	1000	2000
19	2-HG	100	200	500	1000	2000	50
20	Glucose	500	1000	2000	50	100	200
21	AMP	500	1000	2000	50	100	200
22	NAD	1000	2000	50	100	200	500
23	Glucosamine	50	100	200	500	1000	2000

24	UDP	50	100	200	500	1000	2000
25	GTP	100	200	500	1000	2000	50
26	Ribose 5-phosphate	200	500	1000	2000	50	100
27	Histidine	500	1000	2000	50	100	200
28	Cytidine	1000	2000	50	100	200	500
29	Fructose	2000	50	100	200	500	1000

Table S3: Comparison of LOD and LOQ at 0.8 D1 and at 1.5 D1 with and without Gd respectively.

Concentration (μM)	TCI Helium cooled		TXI Nitrogen cooled
	**D1= 0.8 s Gd**	**D1= 1.5 s no Gd**	**D1= 1.5 s no Gd**
**LOD**	7.57	12.61	19.1
**LOQ**	26.89	42.03	65.62
